# Splicing Regulation of Pro-Inflammatory Cytokines and Chemokines: At the Interface of the Neuroendocrine and Immune Systems

**DOI:** 10.3390/biom5032073

**Published:** 2015-09-07

**Authors:** Felitsiya Shakola, Parul Suri, Matteo Ruggiu

**Affiliations:** Department of Biological Sciences, St. John’s University, 8000 Utopia Parkway Queens, Jamaica, NY 11439, USA; E-Mails: felitsiya6@yahoo.com (F.S.); parulsuri281291@gmail.com (P.S.)

**Keywords:** alternative splicing, RNA-binding proteins, inflammation, neurodegeneration, interleukin receptor

## Abstract

Alternative splicing plays a key role in posttranscriptional regulation of gene expression, allowing a single gene to encode multiple protein isoforms. As such, alternative splicing amplifies the coding capacity of the genome enormously, generates protein diversity, and alters protein function. More than 90% of human genes undergo alternative splicing, and alternative splicing is especially prevalent in the nervous and immune systems, tissues where cells need to react swiftly and adapt to changes in the environment through carefully regulated mechanisms of cell differentiation, migration, targeting, and activation. Given its prevalence and complexity, this highly regulated mode of gene expression is prone to be affected by disease. In the following review, we look at how alternative splicing of signaling molecules—cytokines and their receptors—changes in different pathological conditions, from chronic inflammation to neurologic disorders, providing means of functional interaction between the immune and neuroendocrine systems. Switches in alternative splicing patterns can be very dynamic and can produce signaling molecules with distinct or antagonistic functions and localization to different subcellular compartments. This newly discovered link expands our understanding of the biology of immune and neuroendocrine cells, and has the potential to open new windows of opportunity for treatment of neurodegenerative disorders.

## 1. Introduction

Alternative splicing is a versatile regulatory mechanism that can have profound effects on gene expression: almost all polymerase II transcripts undergo alternative pre-mRNA splicing [1], and about 50% of all disease-causing genetic mutations affect pre-mRNA splicing [2,3]. Alternative splicing is a major source of protein complexity in both the immune and nervous systems [4,5,6,7,8,9]. These tissues are cellularly very diverse, comprised of cells that require the processing of large amount of information, ability to adapt, and rapid and precise control of cellular differentiation and activity. Via alternative splicing and nonsense mediated decay (NMD) [10,11,12], gene expression can be down-regulated by creating protein and mRNA isoforms that are unstable or not functional, cytokine signaling can be altered, for example, certain isoforms of cytokines receptors often have antagonistic functions in the signaling of a particular cytokine [13], and protein function can be modulated, for example, alternative splicing of *N*-methyl-d-aspartate receptor 1 [4,14,15] and agrin [16] induces receptor clustering at the postsynaptic membrane.

By changing the coding information of a single gene, alternative splicing can lead to the alteration of the peptide sequence encoded by that gene and often exerts a profound influence on the biochemical properties and biological functions of a given protein [4]. Interestingly, alternative splicing can also affect non-protein-coding genes [17,18]. The process involves generation of distinct mRNA molecules from the same primary transcript by the selective inclusion and/or skipping of specific exons [19,20], and occurs in various patterns, such as differential inclusion or skipping of cassette exons, mutually exclusive exons, competing splice sites and polyadenylation signals. Alongside transcript abundance, alternative splicing can also alter the structure, stability, and turnover of transcripts and the proteins they encode, thus controlling the binding properties, enzymatic activity, protein stability, posttranslational modifications, and intracellular localization of a large number of protein isoforms originating from a single gene [21,22].

The selective inclusion or skipping of exons requires the recognition of *cis*-acting sequences (splice sites; exonic and intronic enhancers and inhibitors) by *trans*-acting factors (small nuclear ribonucleoprotein particles (snRNPs); splicing factors). In humans, there are approximately 200 spliceosomal and splicing-associated proteins which regulate alternative splicing [23,24]. The expression of these factors can be ubiquitous or tissue-specific, or developmentally regulated. It has been demonstrated that the high degree of alternative splicing in the brain correlates with expression of a larger number of splicing regulators than most other tissues [25]. Major examples of alternative splicing factors of the nervous system include the proteins Nova1 and Nova2 [26], and the three Rbfox proteins [27,28,29]. Other alternative splicing factors regulate the cells of the immune system—for example, the alternative splicing factor HuR is essential for the B cell antibody response [30].

**Figure 1:**
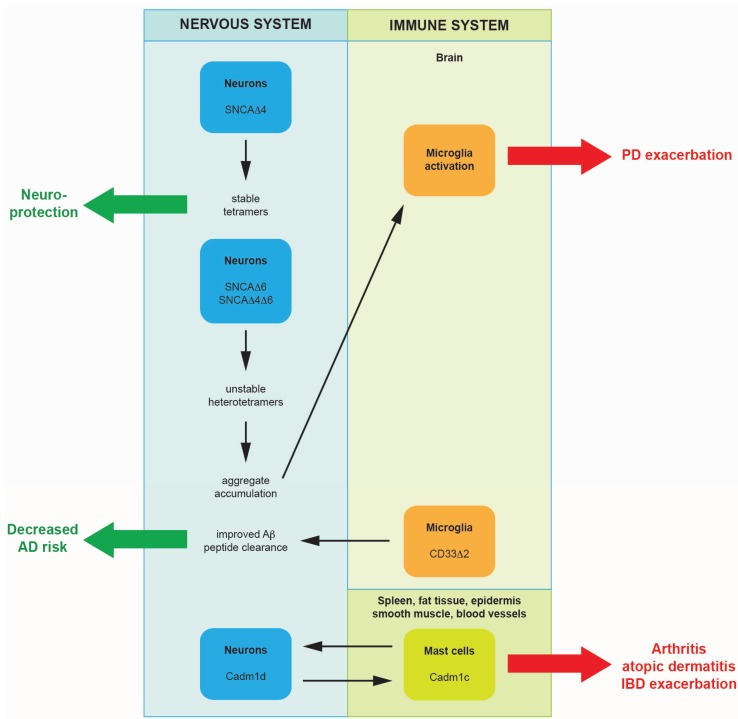
Scheme illustrating the interaction between the nervous and immune systems during inflammation via alternatively spliced isoforms of SNCA, CD33 and Cadm1. Increased expression of particular, shorter SNCA isoforms leads to the formation of unstable heterotetramers that dissociate easily. This results in the accumulation of toxic oligomers, which activate microglia, increasing damage to dopaminergic (DA) neurons, and exacerbating Parkinson’s disease (PD). A non-functional CD33 receptor isoform lacking exon 2 reduces the risk of Alzheimer’s disease (AD) by possibly improving the microglia-mediated clearance of the amyloid-beta peptide (Aβ), thus preventing the formation of amyloid plaques in the brain. Alternative splicing can mediate immune-nervous system communication in inflammation linked to atopic dermatitis, inflammatory bowel disease (IBD), and chronic arthritis, conditions accompanied by neurite outgrowth in the inflamed tissue. The transhomophilic binding of Cadm1, expressed in neurons and in mast cells, brings the immune and nervous system in contact. There is a considerable difference in binding strength among the isoforms that are paired. Peripheral nerves, expressing exclusively CADM1c under physiologic conditions, could change the pattern of CADM1 splicing in pathological conditions, reinforcing nerve-mast cell interaction and exacerbating neurogenic inflammation.

Even though relatively isolated by the blood–brain barrier and with immune privilege, brain tissue is inhabited by various immune cells with different ontogenesis and functions. Under non-pathological conditions, the central nervous system (CNS) houses perivascular, choroid plexus, and meningeal macrophages/dendritic cells (DCs), members of the myeloid lineage, and microglia, the local macrophages, whose population is maintained independently from bone marrow [31]. Perivascular and meningeal macrophages have been suggested to play a role in onset experimental allergic encephalomyelitis, an experimental model for multiple sclerosis (MS) [32]. Meningeal mast cells have been demonstrated to contribute to stroke pathology [33]. Microglia account for 5%–20% of all cells in the CNS [34] and perform not only immune-related functions, but also play a critical role in brain homeostasis, synaptic pruning, adult neurogenesis and learning-dependent synapse formation, the impairment of which leads to neurodevelopmental and neuropsychiatric pathologies [35,36,37]. The lack of conventional lymphatic system of the brain and spinal cord contributes to the term “CNS immune privilege”. However, it has been demonstrated that DCs are able to migrate from the CNS to cervical lymph nodes and preferentially target B-cell follicles rather than T cell-rich areas [38]. Contradicting a previous assumption that T cells cannot detect antigens located within the healthy CNS, it has been demonstrated that T cells activated outside the CNS can detect their antigenic targets even when located within the CNS parenchyma [39], a mechanism important for CNS autoimmunity. Interestingly, the brain can also be infiltrated by tissue-resident memory T cells in the course of inflammation, as observed in MS [40] and schizophrenia [41].

There are several well-characterized examples of splicing isoforms with pro-inflammatory functions, such as interleukin-32 isoforms in rheumatoid arthritis [42], and they have been reviewed elsewhere [43]. In this review, we will explore the communication between the nervous and immune systems via alternatively spliced molecules in the course of inflammation (Figure 1). Alternative splicing in both systems can change as a result of neuroinflammation or of inflammation found elsewhere within the body. We will also use publicly available datasets from next-generation sequencing and high-throughput genome-wide studies to speculate about the factors regulating the expression of specific splice isoforms and their mechanism of action.

Understanding the “talk” between neuroendocrine and immune cells through the language of alternative splicing will provide deeper understanding of the general immune-neuroendocrine system interaction and the mechanisms of signaling during inflammation, and has the potential for the identification of new biomarkers and new avenues of intervention for treating metabolic and neurodegenerative disorders.

## 2. Alternative Splicing Promoting Neurodegeneration or Providing Neuroprotection

### 2.1. Alternatively Spliced Isoforms of Alpha-Synuclein Promote the Progression of Parkinson’s Disease

Many neurodegenerative diseases, including Parkinson’s disease (PD), MS, and Alzheimer’s disease (AD) are associated with chronic inflammation [44,45,46]. Neurodegeneration is characterized by neuronal loss in the presence of inflammation [47], and neurodegenerative disorders appear to share common pathological features represented in the concept of “neuroinflammation”, including activation of microglia and astroglia at the affected brain area (referred to as gliosis), and the subsequent release of pro-inflammatory factors and reactive oxygen species [48]. Numerous studies confirm that chronic inflammation mediated by microglial cells, characteristic for PD, is the crucial factor leading to the death of dopamine (DA)-producing neurons in the brain [49,50,51]. For example, production of inflammatory molecules by microglia often accompanies the slow neurodegenerative process of PD [52]. Misfolded alpha-synuclein protein (SNCA) activates microglia *in vitro*, leading to the secretion of pro-inflammatory cytokines, such as TNF-α [53] and IL-1α [54], and to the production of reactive oxygen species, ultimately damaging DA neurons [55]. Studying early stages of the disease, Alieva *et al.* recently identified significant splicing differences in genes involved in cellular-transport processes in a whole transcriptome study performed on the peripheral blood of untreated patients with stage 1 PD, although no RT-PCR validation was performed [56]. These patient samples display increased amount of shorter, alternatively spliced isoforms of SNCA: increased expression of SNCA isoforms lacking exon 4 leads to the formation of stable tetramers and neuroprotection, while increased expression of SNCA isoforms lacking exon 6 or both 4 and 6 leads to the formation of unstable heterotetramers that dissociate easily, thus resulting in the accumulation of toxic oligomers and increased PD risk [57]. Therefore, alterations in the alternative splicing pattern of SNCA may lead to accumulation of extracellular aggregates of human SNCA, resulting in microglia activation, increased damage to DA neurons, and exacerbation of PD [55]. Little is known about the mechanism regulating alternative splicing of SCNA, but in humans splicing of exon 4 is altered by a E46K mutation on exon 4 itself [57,58], and is also known to be influenced by polyT stretch length [59]. Oxidants induce exon 6 skipping in mice [60]. It is noteworthy that elsewhere exon 4 and 6 are annotated as exon 3 and 5, respectively [61]. Other genes possibly exhibiting differential splicing in the early stages of PD are directly involved in vesicle-mediated transport, and alteration in the transport of synaptic vesicles has been shown in PD [56]. These differentially spliced isoforms should be investigated as possible biomarkers for early onset of PD.

### 2.2. A Single Nucleotide Polymorphism Affecting Alternative Splicing Decreases Levels of Functional CD33 in the Brain: A Mechanism for Providing Protection from Alzheimer’s Disease

Microglia play a role not only in PD, but also in other neurodegenerative disorders, such as AD, Huntington’s diseases, MS, and amyotrophic lateral sclerosis (ALS) [62,63,64]. A specific polymorphism (rs12459419), highly co-inherited with the rs3865444 polymorphism, has recently been identified in the CD33 gene in microglia cells and is known to reduce the risk of AD [65]. CD33 belongs to the sialic acid-binding Ig-superfamily of lectins (SIGLECs)—it mediates cell–cell interaction and inhibits normal functions of immune cells [66]. Interestingly, CD33 levels are increased in the brain of AD patients, and positively correlate with amyloid plaque accumulation and disease severity [67]. It has been shown that high levels of CD33 impair microglia-mediated clearance of the amyloid-beta peptide (Aβ), resulting in the formation of amyloid plaques in the brain [67]. The binding of sialic acid activates CD33, turning down the immune responses of microglia and phagocytosis [68]. A recent study demonstrates that rs12459419 affects exon 2 splicing efficiency and leads to loss of CD33 function, therefore reducing AD risk [65]. Skipping of exon 2 does not alter the reading frame of CD33, but it generates a protein that lacks the IgV domain that typically mediates sialic acid binding in SIGLEC family members; this isoform is suggested to be nonfunctional [65].

### 2.3. A Soluble Isoform of the Interleukin-7 Receptor Increases the Risk of Developing Multiple Sclerosis

MS, a major chronic inflammatory autoimmune disorder of the human CNS, is associated with changes in the splicing patterns of multiple genes, including the interleukin-7 receptor (IL-7R) [69]. IL-7 is a cytokine with a role in the regulation of hematopoiesis, primarily acting on the lymphoid cell lineage [70]. IL-7 supports the survival and growth of precursor B cells [71,72] as well as the growth and maintenance of immature and mature T cells [73,74]. IL-7 is produced by non-hematopoietic stromal cells mostly in the thymus and bone marrow. Alongside its expression in various organs, it is also expressed in the developing brain where it promotes neuronal survival and neurite outgrowth [75]. IL-7 signaling is mediated via its receptor IL-7R. IL-7R is expressed in pre-B cells, in populations of thymocytes, thymic stromal cells, mature T cells, B cells, and in macrophages [76,77,78]. IL-7R and IL-7 are expressed in neuronal progenitor cells [79], as well as in mature human neurons and astrocytes [80]. It has been demonstrated that a single-nucleotide polymorphism of the IL-7R leads to skipping of alternative exon 6. Skipping of exon 6 creates a soluble receptor (IL-7RΔ6), which is associated with an increased risk of developing MS [81,82,83,84]. Both IL-7R and IL-7RΔ6 isoforms have high affinity towards the IL-7 ligand, suggesting that they may have antagonistic functions [85,86]. Interestingly, transmembrane domains are often encoded by a single exon whose skipping generates a soluble version of the receptor [87,88]. The precise mechanism of IL-7RΔ6 action in MS has not been demonstrated. However, the article by Evsyukova *et al.* [69] suggests two hypotheses: First, lower amount of the transmembrane IL7R, due to increased production of IL-7RΔ6, as well as sequestration of IL-7 by IL-7RΔ6 could deprive T cells of their survival and proliferative signals and compromise their survival and proliferation. Increased production of IL-7RΔ6, in fact, lowers the amount of the transmembrane IL-7R. Moreover, sequestration of IL-7 by IL-7RΔ6 could deprive T cells of their survival and proliferative signals and compromise their function by competing with the membrane-bound receptor for the ligand, leading to decreased receptor-mediated signaling, a well-known mechanism of soluble receptor action [88]. This hypothesis is based on studies showing a decrease in CD8^+^ T cells in the CNS, assuming that MS is associated with a decrease in T-cell populations [89]. According to the second hypothesis, lower expression of the full-length IL-7R by CD8^+^ T cells in MS patients increases their susceptibility to viral attack, leading to T-cell “exhaustion” (poor function and viability of memory T cells) and resulting in the autoimmune attack on the myelin sheath typical for MS (for further details, see [69]).

Another example of a leukocyte antigen with a splicing defect reported in patients with MS is CD45 [90,91,92,93]. CD45 is a prototypic receptor tyrosine phosphatase, playing a role in activation and development of T cells [93]. Naive T cells express longer isoforms of the receptor, while activated and memory T cells express shorter isoforms [7,94]. The shorter isoforms bind more efficiently and rapidly form homodimers important for phosphatase activity, implying that this splicing switch is critical for preventing extended T cell receptor signaling and excessive tissue injury [94]. Considering that the longer isoforms are associated with more efficient T cell receptor signaling, failure to attenuate prolonged T cell receptor activation could contribute to the inflammatory response resulting in axon demyelination in MS. The mechanism of alternative splicing of *CD45* has been meticulously characterized in terms of *cis*-acting RNA polymorphism [95,96] and *trans*-acting protein factors [97,98,99,100]. For further details, see [69].

### 2.4. Alternative Splicing of Interleukin-15 Receptor Modulates Interleukin-15 Signaling in Experimentally Induced Neuroinflammation

Interleukin-15 (IL-15) is a well-known T-cell growth factor [101]. It is expressed in various types of cells and tissues—monocytes, macrophages, dendritic cells, keratinocytes, fibroblasts, epidermal skin cells, epithelial cells of various origins and nerve cells [102]. Inflammation from a variety of sources such as scrub typhus, acute pancreatitis, MS, rheumatoid arthritis, *etc*. leads to increased blood levels of IL-15. Peripheral IL-15 then activates multiple signaling pathways in neurons throughout the CNS, and IL-15 receptors (IL-15Rs) show robust upregulation after neuroinflammation [103]. IL-15 signaling shows multi-level regulation of the expression of both the ligand and its specific receptor IL-15Rα, including multiple splice variants, post-translational modifications, and acute regulation of protein turnover. Interestingly, specific IL-15Rα splice variants show differential intracellular localization. In a COS-7 (kidney cells from African green monkey) cell model, the full-length human IL-15Rα is mainly associated with nuclear membrane [104], while deletion of exon 2 leads to retention of the receptor in the endoplasmic reticulum (ER), Golgi, and cytoplasmic vesicles [105]. Isoforms IL-15Rα Δ4, IL-15Rα Δ3 and 4, or IL-15Rα Δ3, 4, and 5 are predominantly associated with the Golgi and ER [106]. Alternative splice variants of IL-15Rα may be important for mediating neuroinflammatory signaling in cerebral endothelia that compose the blood–brain barrier, the interface between the CNS and peripheral circulation that provides protection to the brain from bacterial infections and potential neurotoxins. Mouse brain microvessel endothelial cells express the following receptor isoforms: α1 (IL-15Rα Δ3, 4, 5), α2 (IL-15Rα Δ3, 4, 5 and half of exon 2), α3 (IL-15Rα lacking half of exon 2), α4 (lacking exon 4, but with additional sequence inserted between exons 5 and 6 denoted as exon 5'), and αf encoding the full length IL-15Rα. IL-15Rα shows robust upregulation in response to tumor necrosis factor α (TNF) treatment in rat brain endothelial (RBE)-4 cells, which suggests that the receptor plays a role in neuroinflammation. The mRNA of both αf and α4 is more abundant than that of α1 (least abundant isoform), α2, and α3, but all are increased in response to systemic TNF treatment in microvessels. Most isoforms are present in cytoplasmic vesicles and their trafficking involves the ER and the Golgi complex, but different isoforms have different effect on downsteam IL-15 signaling: α2 and α4 isoforms inhibit basal signal transducer and activator of transcription (STAT3) activation when transfected into RBE4 cells. Therefore, splicing of IL-15Rα appears to be necessary for creating an orchestrated effect in IL-15 signaling, possibly critical for the communication between the brain and other systems during inflammation [104].

### 2.5. Alternatively Spliced Isoforms of the Interleukin-1 Receptor Accessory Protein Promote Neuroprotection during Acute Inflammation of the Central Nervous System

Alternative splicing of certain molecules does not necessarily exacerbate the neurodegenerative effect of acute inflammation in the CNS. Cases are known where an alternatively spliced isoform can act in a neuroprotective manner. It is well known that components of the innate and adaptive immune systems can have antagonistic roles, for example by exacerbating neurotoxicity in neurodegenerative disorders and neuroinflammation, while also having a neuroprotective function through the release of anti-inflammatory mediators and the clearance of toxic compounds [107,108]. A molecule that has the ability to alter interleukin signaling in neurodegeneration by providing neuroprotection by way of changing its pattern of alternative splicing is the interleukin-1 receptor (IL-1R) accessory protein (AcP). The pro-inflammatory, highly active cytokine IL-1 initiates numerous inflammatory and immunological responses to infection, stress, and tissue damage [109] and has multiple functions in both the periphery and the CNS. IL-1 signaling is mediated via a widely expressed complex comprised of the IL-1 receptor (IL-1R) and AcP [110]. A novel isoform of AcP—termed AcPb, contains a different C-terminal exon: the canonical exon 12 is skipped and a novel exon (12b) is utilized, and AcPb expression is restricted to the CNS [111]. The authors of the latter publication demonstrated that AcPb is able to interact with IL-1 and IL-1R but is unable to mediate canonical IL-1 responses: AcPb is unable to recruit adaptor molecules crucial for eliciting IL-1 responses such as MyD88 and the kinase IRAK4. Moreover, unlike AcP, AcPb also does not activate p38, JNK, and ERK in EL4 mouse cell line. The article suggests that AcPb could play an important role in neuroprotection during acute inflammatory response in the CNS, possibly through the regulation of IL-1 activity. Mice lacking AcPb showed intact peripheral IL-1 response and developed experimental autoimmune encephalomyelitis—a peripherally-induced, autoimmune-meditated CNS disease. AcPb-deficient mice were more vulnerable to local inflammatory challenge in the CNS and suffered enhanced neuronal degeneration as compared to AcP-deficient or wild type mice. The results suggest that AcPb could be an additional component of the highly regulated IL-1 system, and it may play a role in modulating CNS responses to IL-1, changing the outcome of inflammation towards neuronal survival [111,112]. Another paper describing AcPb [113] reaches different conclusions about its function—the publication shows that this alternatively spliced variant interacts with MyD88 and transduces the IL-1 signal, activating transcription factor NF-κB and inducing the expression of IL-1-responsive genes. It should be noted that the two groups used different cell lines (mouse EL4 cells in the former, human HEK293T in the latter publication), and that the latter publication includes only cell-line studies and no animal model.

### 2.6. A Soluble Form of the Interleukin-6 Receptor Accelerates Nerve Regeneration after Trauma

Another molecule with a possible neuroprotective role is the soluble interleukin-6 receptor (IL-6R). Cytokines of the interleukin-6 (IL-6) family are involved in the regulation of inflammatory processes of the nervous system. This interleukin family includes: IL-6 (which itself undergoes alternative splicing [114]), ciliary neurotrophic factor (CNTF), and IL-11, the signal of which is mediated through the association of their specific membrane receptors with signal-transducing receptor subunits. Soluble forms of IL-6R (sIL-6R) can be generated by two distinct mechanisms: limited proteolysis [115] or by alternative splicing of the receptor [116]. SIL-6R bound to IL-6 is able to associate with the signal-transducing receptor subunit gp130 [117]. The IL-6/sIL-6R complex then mediates IL-6 function by activating target cells that express gp130 on their cell surface without the need of membrane-bound IL-6R, a mechanism termed *trans*-signaling [118]. *Trans*-signaling is also observed with other IL-6 family members: soluble receptors generated by one cell type bind to their ligands and can initiate downstream signaling through the signal-transducing receptor subunits on different cell types, which do not express the ligand binding receptor subunits. *Trans*-signaling is important for neuronal differentiation and survival responses [119]. Accelerated nerve regeneration after trauma was observed in transgenic mice constitutively expressing human IL-6 and sIL-6R [120]. In wobbler mice, an animal model showing close similarities to ALS [121], coadministration of IL-6 and sIL-6R delays the progression of motor neuron disease [122]; therefore, neurons appear to be sensitive to IL-6 signaling, even though they do not express IL-6R and IL-6 [120,123], and IL-6 signaling in neurons might function through the expression of gp130, a cytokine receptor that interacts with and is activated by the IL-6/sIL-6R complex. The review by März *et al.* connects these studies with upregulated gp130 expression during nerve lesions [124], suggesting that gp130 signaling is important for neuronal survival. The authors of the review propose that some members of the IL-6 family of cytokines may be functionally redundant, in particular IL-6 and CNTF: they both employ gp130 for signaling and have a proven role in neuroprotection. Here, it should be noted that sIL-6R seems to have a beneficial role only in the nervous system—other studies suggest that IL-6 trans-signaling via the soluble IL-6R is pro-inflammatory in models of inflammatory bowel disease (IBD), peritonitis, rheumatoid arthritis, atherosclerosis pancreatitis, colon cancer, ovarian cancer and pancreatic cancer, and therefore soluble gp130Fc (gp130 fused with Fc, a constant region of an immunoglobulin heavy chain) is considered a possible therapeutic agent for the treatment of chronic inflammatory diseases and cancer [114].

## 3. Changes in Alternative Splicing of the Neuroendocrine System as a Response to Inflammation Elsewhere in the Body

### 3.1. Alternative Splicing of Interleukin-5 Receptor α Changes in Systemic Inflammation, Possibly Leading to Alterations in Neuronal Growth and Function

The brain appears to be able to respond to inflammation taking place elsewhere in the body by changing the alternative splicing profile of specific genes such as IL-5R [125]. This in turn could have an influence on neuronal structure, function, and signaling, providing an adequate or insufficient/superfluous response to the presence of pathogens or to sterile inflammation, the stimuli of which include mechanical trauma, body’s own cells (in ischemia) and body’s own proteins (in AD and autoimmune disease), and environmental toxins, minerals, crystals, and chemicals [126]. With the onset of inflammation the brain changes the alternative splicing profile of the interleukin-5 receptor α (IL-5Rα), in order to perceive and react to the inflammatory signals of interleukin-5 (IL-5). IL-5 is known to be a late differentiation factor for eosinophils and B-lymphocytes [127]. It is constantly expressed throughout development, while the ligand-specific IL-5Rα is tightly regulated in its expression particularly in bone marrow cells—one of the most critical issues in eosinophil lineage commitment [128], including regulation by alternative splicing [129]. IL-5 and IL-5Rα are not expressed in neurons, but in astrocytes—the largest population of cells in the vertebrate CNS, important for neuroimmunology and neurotransmission and highly heterogeneous in morphology and function. The pattern of alternative splicing of IL-5Rα changes during development in mice—from expression of the transmembrane receptor and five of its alternatively spliced isoforms in embryonic tissue to one soluble isoform immediately after birth—making the brain completely unresponsive to IL-5 signaling, to no expression of any IL-5Rα isoform in adult mice. However, expression of all isoforms resumes in an experimental systemic inflammatory reaction to ovalbumin and schistosomal infection associated with elevated systemic levels of IL-5. Since both IL-5 and IL-5Rα are produced in astrocytes, it has been suggested that IL-5 has specific autocrine and/or paracrine function in those cells [130]. Together with IL-4, IL-5 induces nerve growth factor production in astrocytes, without changes of neuron morphology, possibly with alterations of neuronal function [131]. Splicing of IL-5Rα in astrocytes could have a functional role in their communication with neurons, resulting in reaction of the nervous system to inflammation. Moreover, functional difference between the transmembrane and the soluble IL-5Rα isoforms has also been demonstrated in airway eosinophils [132]. While the membrane-bound IL-5Rα isoform promotes IL-5-mediated growth and antiapoptotic signals to eosinophils (human [132]), the soluble receptor isoform can bind and neutralize IL-5, possibly through preventing interaction of the cytokine with the membrane-bound IL-5Rα isoform (mouse [133,134]). Here, it should be noted that the pattern of alternative splicing of IL-5Rα in mice and humans is different—humans have only one soluble isoform-specific exon, which when included leads to production of a soluble receptor, and when skipped to production of the transmembrane isoform; mice, on the contrary, have a transmembrane isoform-specific exon—skipping produces soluble isoforms, inclusion produces one transmembrane isoform [130]. Exon 9 in mice encodes the transmembrane domain [135]. Antisense oligonucleotides (ASOs) designed to bind with high affinity to the 3'-splice sites of the introns preceding exon 9 IL-5Rα pre-mRNA result in specific deletion of exon 9 and inhibition of the membrane isoform expression in mice [136]. In humans, in-frame stop codons in exons 10 and 11 produce soluble forms of the receptor in the absence of alternative skipping [129,137,138]. It has been suggested that targeting the exons downstream of exon 10 with RNase H-dependent oligonucleotides in patients could lead to the activation of RNase H, hydrolization of the RNA strand of the RNA/oligonucleotide duplexes, and production of soluble IL-5Rα. This, in turn, would neutralize IL-5 and inhibit eosinophil activation. This ASO-mediated strategy to redirect RNA splicing has potential therapeutic utility in asthma and other eosinophilic diseases [136].

### 3.2. Alternatively Spliced Isoforms of Cell Adhesion Molecule-1 Could Reinforce Nerve-Mast Cell Interaction and May Exacerbate Neurogenic Inflammation

Neurite outgrowth is observed in inflamed tissue in many autoimmune diseases such as atopic dermatitis [139,140], IBD [141,142,143], chronic arthritis [144,145], as well as during acute inflammation in myocardial infarction [146], and interstitial cystitis [147]. Thus, it is possible that the peripheral nervous system may communicate directly with the immune system—mast cells localize around nerves and can be in contact with nerve fibers [147]. The close proximity of nerve and mast cells is considered a functional unit of neuro-immune mechanisms and this interaction is sustained by transhomophilic (with another molecule of the same kind) binding of cell adhesion molecule-1 (CADM1), a member of the Ig superfamily [148]. CADM1 is alternatively spliced: in humans and mice there are four membrane-spanning isoforms, CADM1a to d, with different lengths of the region upstream of the transmembrane domain—CADM1a includes all known exons, CADM1b skips exon 8, CADM1c skips exon 9, and CADM1d skips both exon 8 and 9. CADM1 changes its alternative splicing pattern during development of the mouse cerebrum leaving CADM1d as the predominantly expressed isoforms in mature cerebrum, and this isoform enhances nerve-mast cell interaction [148]. Neurons express different combinations of the four CADM1 isoforms [149], while mast cells in mice express only CADM1c [150,151]. The homophilic binding strength differs considerably among the isoforms that are paired with CADM1c in *trans* interaction [152]. Therefore, CADM1 splicing in neurons may be involved in the regulation of nerve-mast cell interaction, which has a role in inflammatory diseases such as atopic dermatitis, alopecia, asthma, and IBD [153,154,155]. Peripheral nerves, which express exclusively CADM1c under physiologic conditions [156], may change the pattern of CADM1 splicing in pathological conditions, and this in turn could reinforce nerve-mast cell interactions and exacerbate neurogenic inflammation [148]. It has been also hypothesized that another alternatively spliced variant of the same molecule—soluble CADM1, might be involved in directional neurite extension by serving as an anchor to which membrane-bound CADM1 on the neurites can bind [149]. It is important to note here that there are major phenotypic and functional differences between human and mouse mast cells [157]: human mast cells express four functional isoforms (SP1, SP3, SP4, and SP6) with different roles in survival and homotypic adhesion and two non-functional (c15, c450) alternatively spliced CADM1 isoforms [158], but up to this date there is no available information on alternatively spliced CADM1 isoforms expressed in neurons in humans.

### 3.3. An Alternatively Spliced Isoform of the Thyroid Stimulating Hormone Produced during Acute and Chronic Inflammation May Alter Metabolism by Interfering with the Native Hormone Signaling

Alternative splicing may also be involved in the communication between the immune and endocrine systems during inflammation by interfering with the thyroid stimulating hormone (TSH)-signaling from the brain that regulates whole-body metabolism. TSH has an altered isoform ratio during inflammation. This hormone is produced by the anterior pituitary and primarily acts on the thyroid gland to produce thyroxine (T4), the prohormone of triiodothyronine (T3), essential for the control of metabolic activity, growth, mood and cognition. TSH is composed of noncovalently bound TSHα and TSHβ subunits, where the TSHβ subunit confers the immunogenic and hormonal functions [159]. Interestingly, the pituitary is not the only source of TSH used by the thyroid: it is also produced by lymphocytes [160,161]. Immune system-derived TSH has a well-known role of a cytokine-like molecule during growth and differentiation of hematopoietic cells [162], with its primary action directed to the thyroid itself [163]. An alternatively spliced isoform of the TSHβ subunit named TSHβv may influence the T3 and T4 secretion during infection or inflammatory-related thyroid disorders [164]. TSHβv is found in mice and it is expressed in the pituitary, in peripheral blood leukocytes (PBL), and in the thyroid. It retains a part of intron 4 and lacks exon 4, retaining the region that interacts with TSHα and mediates dimerization [165]. Reovirus infection results in a sevenfold increase in TSHβv expression, while TSHβ remains unchanged [166]. High TSHβv levels are associated not only with acute, but also with chronic inflammation: in a study of patients with Hashimoto’s thyroiditis (HT), TSHβv transcript levels were elevated in PBL of HT patients compared to normal controls [165]. This observation was reversed by prednisone treatment of HT patients having a short duration of illness (≤9 months) compared to patients with a long duration (≥18 months) or to controls.

There are several hypotheses explaining how TSHβv might regulate thyroid hormone activity. For example, it could function as an agonist to elicit a thyroid hormone response leading to increases in T3 and T4 synthesis and upregulation of metabolic activity. TSHβv, in fact, retains exon 5, which is important for the biological function of TSH as it includes an 18-amino-acid region used for attachment to the α-subunit. Therefore, TSHβv may retain the ability to function in a heterodimeric complex TSHα, and TSHβ remains functionally active in terms of intracellular signaling. This is supported by evidence showing that TSHβv induces a cAMP response from mouse alveolar macrophage cells and rat thyroid FRTL-5 cells [166]. A second, conflicting hypothesis suggests that TSHβv could also have an antagonistic activity: by binding to and competing for TSH receptor signaling, TSHβv may restrict thyroid hormone synthesis and this, in turn, may lead to lower circulating T4 levels and lower host metabolic functions by blocking native TSHβ binding [167]. This suggests a more complex pattern of microregulation of thyroid hormone activity by immune system TSH, which could be important for adjusting metabolism during periods requiring energy conservation [166].

## 4. Regulation of Alternative Splicing at the Interface of the Immune and Neuroendocrine Systems during Inflammation

**Figure 2:**
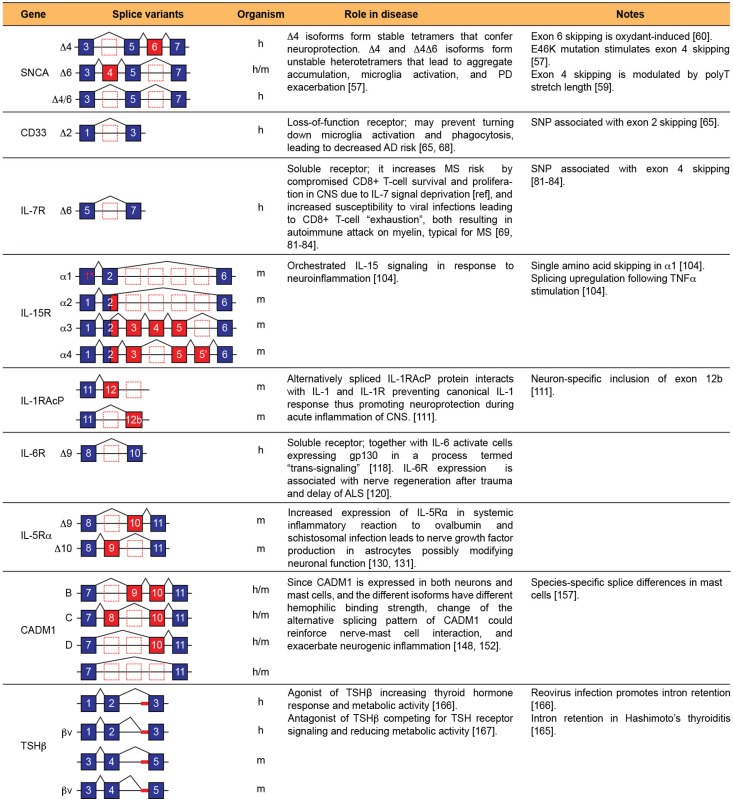
Alternatively spliced isoforms mediating communication between the nervous system and the immune system during inflammation, and their capacity to alter disease outcome. The organism in which the splice forms have been characterized is indicated (h: human; m: mouse).

In this review, we have described alternatively spliced isoforms of pro-inflammatory genes that differ functionally and in their subcellular localizations, and the roles they play in both the neuroendocrine and immune system, both in normal physiological conditions and in disease (Figure 2). However, despite their central role in inflammation, neurodegeneration, and neuroprotection, very little is known not only about the function of many, if not most, such splice isoforms, but also what splicing factors and/or RNA-binding proteins (RBPs) regulate their splicing and how.

The advent of next-generation techniques to study RNA-protein interactions such as UV cross-linking and immunoprecipitation (CLIP [168]), and direct sequencing of the entire transcriptome (RNA-Seq [169,170,171]), or a combination of high throughput sequencing and CLIP (HiTS CLIP [172], or CLIP-Seq [173]), has allowed scientists interested in gene regulation to gradually shift their focus from the study of transcription factor-DNA interactions to a new one focusing on RNA regulation [174]. CLIP in particular is an extremely powerful technique: not only it allows the detection and identification of transcripts directly interacting with a specific RBP in living organisms, but it also allows the identification of the binding site of that protein on each transcript to which that protein is bound on a genome-wide scale and at a single nucleotide resolution [175]. Knowing the binding site of an RBP onto a transcript in turn allows researchers to design testable hypotheses regarding the mechanism of regulation.

We therefore decided to mine publicly available CLIP and RNA-seq datasets for known regulators of alternative splicing, including Rbfox2 [176], Rbfox3 [177], hnRNP C [178], hnRNP L [179,180], Nova [181], PTB [182], HuR [183], and SRSF3 and SRSF4 [184]. By analyzing previously published data, we asked whether any of the above-mentioned splicing regulators may be involved in controlling alternative splicing of the genes that we described earlier in this review (SNCA, CD33, IL-5R, IL-6R, IL-7R, IL-15R, IL-1RAcP, CADM1, and TSHB). We first asked whether any of the genes that we describe in this review appears in published high-throughput functional studies of the above-mentioned RBPs. We then uploaded publicly available CLIP datasets onto the UCSC Genome Browser and looked for CLIP tag clusters in the proximity of (*i.e.*, within surrounding introns), or overlapping with, alternatively spliced exons. By doing so we were able to identify 5 RBPs as putative regulators of some of the splicing events described in this review: Nova, HuR, hnRNP L, PTB, and hnRNP C.

**Figure 3:**
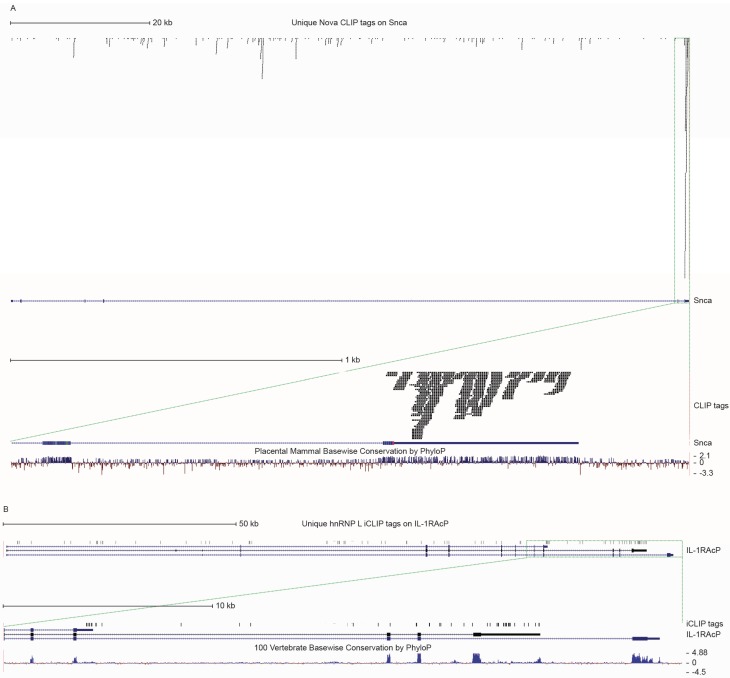
Proposed approach for the identification of splicing factors and RBPs that may regulate alternative splicing of specific pro-inflammatory genes and their binding sites onto the pre-mRNA. Publicly available CLIP datasets have been uploaded as custom tracks on the UCSC Genome Browser (http://genome.ucsc.edu/). For each gene, thick bars represent exons, while thin bars represent introns. Arrowheads along introns indicate the direction of transcription. The panel on top shows the entire genomic region, while the panel at the bottom shows where CLIP clusters are located. CLIP tags are indicated as bars. (**A**) Nova CLIP tags on the Snca gene on mouse chromosome 6 (assembly: mm9). Nova binds to Snca pre-mRNA in mouse brain, with robust CLIP tag clusters in the 3' UTR; (**B**) hnRNP L iCLIP tags on the IL-1RAcP gene on human chromosome 3 (assembly: hg19). HnRNP L binds to human IL-1RAcP pre-mRNA in HeLa cells. IL-1RAcP utilizes three different 3' UTR-encoding exons; interestingly, hnRNP L CLIP clusters are localized on the two more proximal 3' UTRs, while no clusters are detected on the more distal one. CLIP tags coming from the plus strand are shown in blue, while CLIP tags coming from the minus strand are shown in red.

NOVA proteins are neuron-specific antigens targeted in the autoimmune disorder paraneoplastic opsoclonus myoclonus ataxia (POMA), an autoimmune neurologic disease characterized by abnormal motor inhibition [185,186]. Nova proteins regulate neuronal pre-mRNA splicing by directly binding to RNA in a sequence-specific manner [168,187,188]. By using a HiTS-CLIP dataset (a particularly robust dataset, consisting of more than 80 million CLIP tags from 20 individual experiments [172]), our analysis suggests that three of the cytokine genes described in this review may be regulated by Nova: SNCA (with CLIP clusters on the 3' UTR; Figure 3A), IL-1RAcP (with CLIP clusters surrounding exon 12); and CADM1 (with CLIP clusters in the proximity of exon 8).

HuR (ELAVL1) is an essential and ubiquitous protein member of the ELAV/Hu family of RBPs, necessary for proper embryonic development and immune response [189,190]. All mammalian members of the ELAV/Hu family, including HuR, positively regulate stability and translation of target mRNAs [191,192,193]. Using a PAR (photoactivatable ribonucleoside)-CLIP [194] dataset [195], HuR binding sites were identified on the pre-mRNAs of SNCA, IL-6R, CADM1, and IL-1RAcP. IL-1RAcP is particularly interesting, since both HuR and Nova CLIP tag clusters are located on the 3' UTR, which is encoded by exon 12, suggesting that perhaps this transcript maybe co-regulated by Nova and HuR.

Heterogeneous nuclear ribonucleoprotein L (hnRNP L) is a multifunctional RBP that contains four RNA-recognition motifs (RRMs) and targets specific CA-repeat and CA-rich RNA elements. It has wide-ranging functions on mRNA metabolism, such as export of intronless mRNAs, regulation of translation and mRNA stability, poly(A) site selection, and alternative splicing [179]. By using a iCLIP whole genome dataset [179], CLIP tags were identified on SNCA (with iCLIP tags spread throughout the whole gene, and especially on 3' UTR exon 6) IL-1RAcP (with iCLIP tags on whole gene, especially at 3' UTR after alternatively spliced exon 12; Figure 3B), IL-6R (with iCLIP tags on whole gene, especially around alternatively spliced exon 9), IL-5R (with iCLIP tags upstream of alternatively spliced exon 11), IL-15R (with iCLIP tags downstream of alternatively spliced exon 3 and over exon 4), and CADM1 (with iCLIP tags on the whole gene, especially around alternatively spliced exon 8, 9, and 10).

Polypyrimidine tract binding protein (PTB; also known as hnRNP I) is a well-characterized splicing suppressor [196]. PTB binds to CU-rich elements, often overlapping with the U2AF65 binding sites near the 3' splice site [182]. Using publicly available CLIP-seq whole genome data [182], SNCA appears to have several PTB CLIP tags in intron 4 upstream of alternatively spliced exon 5.

In summary, using publicly available CLIP and RNA-seq datasets, we can hypothesize that SNCA may be co-regulated by PTB, Nova, and hnRNP L, with perhaps PTB regulating alternative splicing, and Nova and hnRNP L mediating RNA stability via binding in the 3' UTR (Figure 3A). It is worth noting here that the situation with SNCA is complex, since this gene does not appear to undergo alternative splicing in the mouse, while exon 4 and exon 6 are alternatively spliced in humans. At the same time, available Nova CLIP data have been obtained from mouse tissue, but not human samples. Similarly, Nova may regulate alternative splicing of Cadm1 while hnRNP L may control RNA stability by binding in its 3' UTR. Finally, IL-1RAcP may be co-regulated by HuR, Nova, and hnRNP L, with Nova mediating alternative splicing regulation, whereas hnRNP L and HuR binding in the 3' UTR may control RNA stability (Figure 3B). Given that the RBPs here described bind RNA in a sequence-specific manner, and given that for many genes knock out mouse models (for example in the case of Nova [16,197,198]) and/or knock down cell lines (for example, in the case of hnRNP L [179,180]) have been generated and used to explore global splicing patterns, it will be possible to test whether specific genes are truly downstream targets of these splicing factors by designing cell-based minigene splicing assays and by mutating the putative binding site(s) identified through database analysis as we just described [199]. Moreover, CLIP and RNAseq experiments designed to compare splicing patterns in normal *versus* inflammation states should also be highly informative [200,201,202].

## 5. Conclusions

Alternative splicing has a tremendous capacity to generate proteins with different structure and functions, allowing for both dramatic and finely tuned communication between different systems within the body. As such, it has potential for exacerbating or alleviating neurological disorders like PD, AD, MS, and acute inflammation both in the CNS and in other tissues, such as atopic dermatitis, IBD, chronic arthritis, *etc.* Better knowledge of the mechanisms of alternative splicing at the interface of the nervous and immune systems could provide biomarkers for diagnosis of early stages of disease, while at the same time specific splice variants may represent new entry points for therapeutic intervention. A first step towards understanding how these mechanisms are regulated is to identify the splicing factors necessary for the generation of specific splice isoform and their binding sites on the pre-mRNA of that particular gene. This can be achieved by designing splicing reporter minigene assays for dissection of the alternative splicing mechanism in cell lines [203,204,205] and methods aimed at altering the splicing outcome for particular exons, ensuring targeted treatment and diagnostics.
